# Recent advances in tumor targeted polymeric nanoparticles for HNC treatment: Enhancing therapeutic efficacy via engineered and biocompatible drug delivery systems

**DOI:** 10.1016/j.jobcr.2025.08.012

**Published:** 2025-08-19

**Authors:** Manoj Kumar Srinivasan, Monisha Prasad

**Affiliations:** a Molecular Biology Lab, Department of ENT, Saveetha Medical College, Saveetha Institute of Medical and Technical Science, Chennai, Tamil Nadu, India; b Molecular Nutrition and Genomics Lab, Department of Community Medicine, Saveetha Medical College and Hospital, Saveetha Institute of Medical and Technical Sciences, Chennai, Tamil Nadu, India

**Keywords:** Head and neck cancer, Polymeric nanoparticles, Chitosan, Cyclodextrin, poly(ethylene glycol)

## Abstract

Head and neck cancer (HNC) represents a heterogeneous group of malignancies with high global incidence and mortality rates. Traditional treatments for advanced HNC, such as combined radiotherapy and chemotherapy, often lead to debilitating long-term side effects that significantly affect patients quality of life. In response to these limitations, tumor-targeted polymer-based nanoparticles have emerged as a promising strategy to improve therapeutic efficacy while minimizing adverse effects. Polymeric nanoparticles comprised of natural or synthetic polymers and sized between 10 and 1000 nm have several benefits, including biocompatibility, adaptable drug release patterns, and the capacity to improve the solubility and stability of medicinal compounds. These nanoparticles can be engineered to target medications to tumor sites specifically, lowering systemic toxicity and enhancing treatment results. This review focuses on recent advances in the targeted therapy of HNC using polymeric nanoparticles such as chitosan, alginate, cyclodextrin, poly (lactic acid), poly (caprolactone), poly (ethylene glycol), dendrimers, and micelles. The emphasis is on their mechanisms, advantages, and potential to transform present HNC treatments.

## 1 Introduction

Head and neck cancer (HNC) is a prevalent malignancy, with the GLOBOCAN reporting 946,456 new cases and 482,001 deaths in 2022, of which nearly 90 % are head and neck squamous cell carcinoma (HNSCC) cases.^1^, ^2^, ^3^ By 2040, the incidence of HNSCC is projected to increase by 40 %, with over 600,000 new cases annually.^4^ The rising prevalence of human papillomavirus (HPV), especially high-risk types 16 and 18, is linked to increasing HNC cases in younger individuals. Additional risk factors include tobacco use, alcohol consumption, oral sex, radiation exposure, chronic mechanical irritation, and infections with Epstein–Barr virus (EBV) and hepatitis B virus (HBV).^5^, ^6^, ^7^, ^8^ However, public health initiatives like HPV vaccination, reduced tobacco use, and early detection through precision diagnostics are expected to reduce incidence by 2060.^3^^,^^6^^,^^9^ HNSCC arises from mucosal linings and is driven by both external carcinogens and genetic alterations (e.g., Fanconi Anaemia Complementation Group [FANC], Grainyhead-like 3 [GRHL3], Filaggrin [FLG], Y-box binding protein 1 [YBX1]).^10^, ^11^, ^12^, ^13^ Oropharyngeal cancers are increasingly HPV-driven, with projections indicating HPV-positive cases may predominate in the near future.^14^^,^^15^

Chemotherapy remains the primary approach for treating various cancers, often administered alone or in combination with other chemotherapeutic agents or plant-derived compounds. Nonetheless, it is associated with several limitations, including significant systemic side effects, poor tumor penetration, and suboptimal pharmacokinetics. Additionally, the emergence of multidrug resistance in some chemotherapeutic regimens poses a major challenge to effective treatment.^16^ Likewise, many phytoconstituents face challenges such as low solubility and poor stability, often requiring high doses to reach therapeutic efficacy.^17^ To address these limitations, nanotechnology has been increasingly explored for HNC therapies, offering enhanced treatment efficacy and minimized side effects.^18^ Currently, nanoparticles are widely utilized in cancer treatments due to their targeted drug delivery systems in photothermal therapy, photodynamic therapy (PTD), immunotherapy, and chemotherapy.^19^

Recent research has explored various nanoparticle-based platforms for targeted cancer drug delivery, including systems composed of lipids, inorganic materials, and polymers, either by encapsulating or conjugating therapeutic agents.^20^ In particular, there has been a significant increase in the development of polymeric nanoparticles over the past few years, aimed at improving targeted drug delivery strategies for HNCs. These advancements are primarily intended to address challenges such as the difficult localization of tumor cells and to minimize adverse effects on surrounding healthy tissues.^21^

Polymeric nanoparticles are typically very small, with sizes generally ranging from 1 to 1000 nm.^22^ Based on their fabrication technique, these nanoparticles can form either nanocapsules or nanospheres. Nanocapsules consist of a drug-containing core enclosed by a polymer shell, whereas in nanospheres, the therapeutic compound is uniformly dispersed within a cross-linked polymer matrix. Additionally, in both structural types, drugs may also be adsorbed onto the surface of the nanoparticles.^22^^,^^23^

Biodegradable and biocompatible polymers are frequently employed in the fabrication of polymeric nanoparticles. These materials include synthetic alternatives such as poly (lactic acid) (PLA) and poly (caprolactone) (PCL), as well as copolymers such as poly (lactic-co-glycolic acid) (PLG), which have been authorized for pharmaceutical use by the United States FDA.^22^, ^23^, ^24^, ^25^ Furthermore, natural polymers such as chitosan and collagen are often employed in polymer-based drug delivery systems due to their low immunogenicity, which permits the carrier matrix to degrade into non-toxic byproducts upon drug release.

A key benefit of polymeric nanoparticles is the ability to tailor their characteristics by modifying the base polymer. This customization enables controlled drug release at targeted sites within the body and enhances the solubility of hydrophilic drugs. Furthermore, encapsulating drugs with short biological half-lives in nanoparticles can help extend their therapeutic presence.^25^ The purpose of this study is to investigate current advances in polymer-based nanoparticles for the targeted therapy of HNC. It emphasizes their ability to improve treatment efficacy while decreasing negative effects associated with conventional medicines.

The discussion focuses on various natural and synthetic polymers used in nanoparticle formulations. Emphasis is placed on their mechanisms, benefits, and potential to transform current HNC treatment strategies.

## 2 Polymeric nanoparticles

### 2.1 Composition and functional role of polymers

Polymeric nanoparticles are fundamentally composed of polymers, surfactants, and an aqueous medium. Among these, polymers serve as the structural foundation due to their macromolecular nature, consisting of repeating monomeric units. The application of nanocarriers in drug delivery has been significantly advanced by enhancing key drug characteristics such as solubility, targeting efficiency, release profiles, pharmacokinetics, and pharmacodynamics. As such, polymers are pivotal in the formulation of polymeric nanoparticles. Therefore, researchers need to have a thorough understanding of polymer attributes such as biocompatibility, biodegradability, chemical stability, drug interaction potential, glass transition temperature, permeability, and overall safety before formulation. To tailor the properties of these nanocarriers, including their release kinetics, target specificity, and biological compatibility, polymer functionalization is a central strategy. This customization can be accomplished through chemical modifications, surface conjugation with targeting ligands, or the integration of lipids to meet specific therapeutic goals.^26^

### 2.2 Classification and structural considerations

**Fig. 1:**
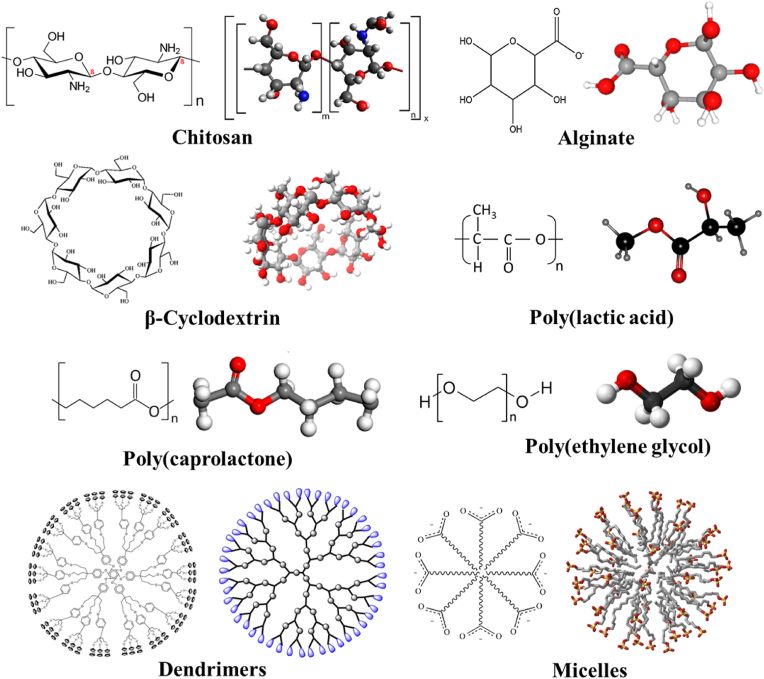
Chemical structures and 3D models of major polymers used in nanoparticle drug delivery (chitosan, alginate, β-cyclodextrin, PLA, PCL, PEG, dendrimers and micelles).

Polymers are broadly classified into two types based on their origin: natural and synthetic polymers. Their structural organization varies with their content and mode of growth. Active substances might be encapsulated within the nanocapsule's core or attached to the polymer's surface. In contrast, active agents in nanospheres might be incorporated in the matrix or adsorbed onto the polymer's surface.^27^ The primary polymers utilized in nanoparticle-based drug delivery for HNC, such as chitosan, alginate, β-cyclodextrin, PLA, PCL, PEG, dendrimers and micelles, are illustrated in Fig. 1.

### 2.3 Mechanisms of targeting and drug release in HNC

Various polymeric nanoparticles such as chitosan, alginate, cyclodextrin, dendrimers, PLA, PLGA, PEG, and micelles have been extensively explored for drug delivery applications in HNC. Following administration, nanoparticles circulate in the bloodstream, where PEGylation enhances their stability and evades reticuloendothelial system (RES) clearance. These nanoparticles accumulate at the tumor site through passive targeting via the enhanced permeability and retention (EPR) effect or via active targeting mechanisms utilizing ligands that bind to overexpressed receptors on HNC cells (EGFR, CD44).^26^

**Fig. 2:**
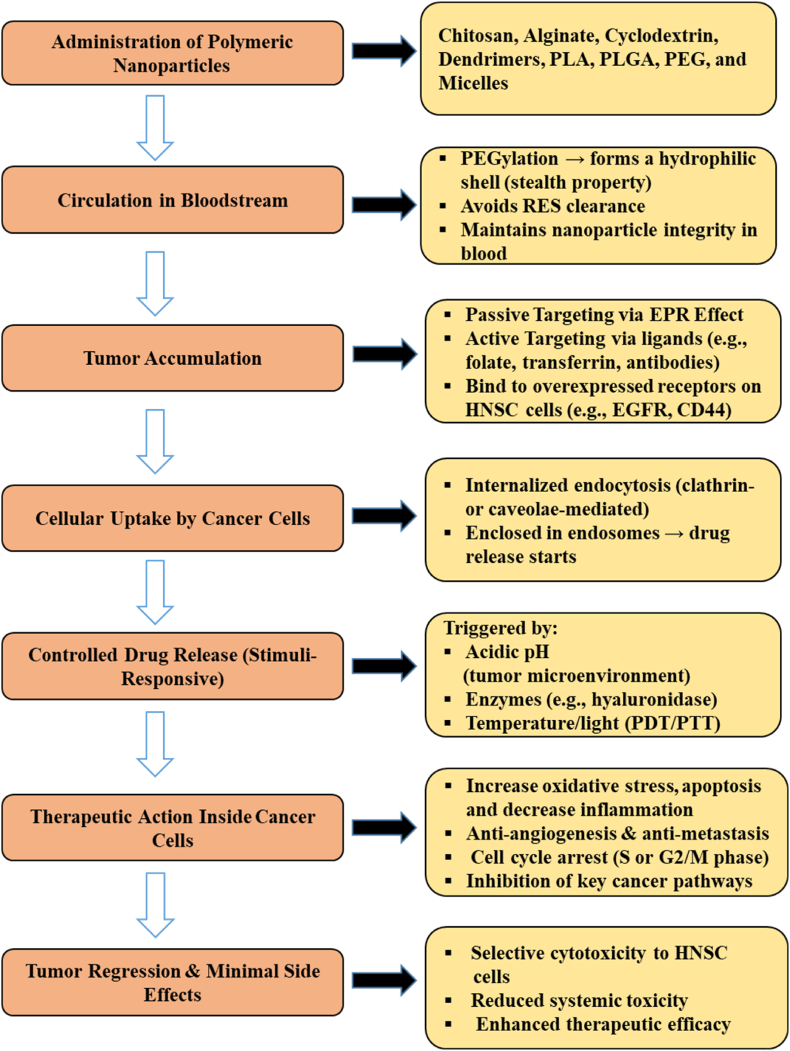
Schematic representation of the mechanism and advantages of polymeric nanoparticles in the treatment of head and neck squamous cell carcinoma.

Upon internalization by cancer cells through endocytosis, stimuli-responsive drug release is triggered by acidic pH, specific enzymes, or external stimuli such as light or temperature. The released therapeutic agents act intracellularly to induce apoptosis, inhibit angiogenesis and metastasis, cause cell cycle arrest, and suppress oncogenic pathways. This ultimately leads to tumor regression with minimal side effects, offering selective cytotoxicity towards HNC cells, reduced systemic toxicity, and improved therapeutic outcomes.^25^^,^^26^ The therapeutic actions and advantages of polymeric nanoparticles in HNSCC treatment are depicted in Fig. 2.

## 3 Chitosan and alginate nanoparticles on HNC

Chitosan and alginate are natural biopolymers that are commonly employed in the production of nanoparticles for medication delivery and therapeutic purposes.^28^^,^^29^ Chitosan is formed from chitin, which is typically found in the exoskeletons of crustaceans such as shrimp and crabs, and is made up of repeated glucosamine units.^30^ Chitosan, a cationic mucoadhesive polymer, enhances bioavailability with saturation uptake (∼2 h residence time, degradation ∼0.7 h^−1^), primarily via clathrin-mediated endocytosis. Drug release occurs through swelling, diffusion, and erosion, with enzymatic lysosomal degradation leading to 60–70 % exocytosis in 24 h. Alginate, on the other hand, is extracted from brown seaweed and consists of alternating blocks of mannuronic and guluronic acid.^31^ Alginate, an anionic polymer, achieves prolonged circulation when surface-modified and is internalized via endocytosis, releasing drugs by diffusion and swelling under acidic or ion-exchange conditions. It degrades via lyases or hydrolysis, clearing through renal or RES pathways. These two polymers interact electrostatically, chitosan being positively charged and alginate negatively charged enabling the formation of stable nanoparticles through ionic gelation. The resulting structure often features a core-shell architecture, with chitosan typically forming the outer layer due to its mucoadhesive properties. This biocompatible and biodegradable nanoparticle system is highly suitable for encapsulating various therapeutic agents.^32^^,^^33^

### 3.1 Chitosan and alginate nanoparticles for 5-FU delivery in HNSCC

A new 5-fluorouracil (5-FU) delivery system was designed combining chitosan (CS) and polycaprolactone (PCL) microparticles (MPs), resulting in a high 5-FU entrapment efficiency (38.57 %) and sustained drug release over 96 h. *In vitro* investigations with human HNSCC cell lines (CAL27 and HSC3) and a preclinical mouse model (AT84) revealed a considerable reduction of cancer cell growth and colony formation while preserving normal fibroblast cells. The composite system also induced autophagy and apoptosis, as shown by elevated LC3-II expression and PARP1 cleavage. These outcomes indicate that CS-PCL MPs may enhance the therapeutic efficacy of 5-FU in treating HNSCC. Furthermore, composite sponges with higher CS concentrations exhibited greater suppression of tumor cell growth and motility. The selective cytotoxicity of CS towards malignant cells was attributed to its positive charge, which promotes interaction with cancer cell membranes. CS also appears to affect cell death pathways and has anti-metastatic properties. Overall, the findings highlight the potential of CS-decorated PCL MPs as a drug delivery platform for HNSCC.^34^

### 3.2 Erlotinib–curcumin co-delivery via chitosan-based hydrogel

PLGA nanoparticles loaded with Erlotinib (Er) and Curcumin (*Cm*) were created to address solubility and bioavailability issues in the treatment of HNSCC. The optimized nanoparticles (optEr/Cm-NP) were incorporated into a chitosan/β-glycerophosphate hydrogel (HG), resulting in an injectable intratumoral delivery method. This formulation displayed desirable physicochemical properties, such as prolonged drug release and increased mechanical stability. *In vitro* cytotoxicity experiments on the FaDu HNSCC cell line revealed that optEr/Cm-NP and its hydrogel formulation were more effective than free medicines. Cellular uptake experiments showed that medicinal drugs were more effectively internalized when administered via the NP or NP-HG system. Furthermore, apoptotic assays showed a considerable increase in cancer cell death. *In vivo* investigations in a xenograft mouse model demonstrated the therapeutic effectiveness of the intratumorally injected optEr/Cm-NP-HG21, which dramatically suppressed tumour development.^35^

### 3.3 Chitosan-coated nanoliposomes for docetaxel delivery

**Fig. 3:**
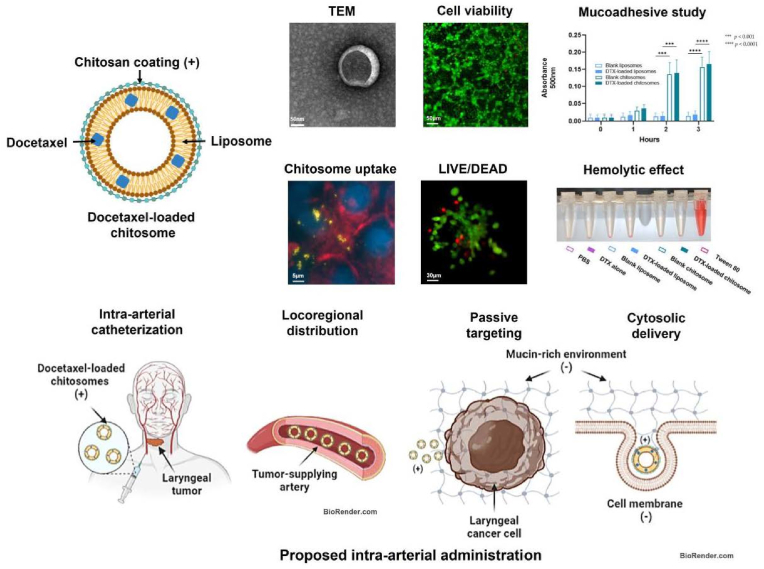
Schematic and experimental analysis of docetaxel-loaded chitosomes for targeted intra-arterial delivery in laryngeal cancer^36^.

Moya-Garcia et al. (2023) developed and evaluated docetaxel-loaded anionic nanoliposomes coated with mucoadhesive chitosan (chitosomes) for chemotherapeutic drug delivery. Anionic liposomes were 99.4 ± 1.5 nm in diameter and had a zeta potential of −26 ± 2.0 mV. Chitosan coating raised the size to 120 ± 2.2 nm and surface charge to 24.8 ± 2.6 mV. Chitosome formation was established using FTIR spectroscopy and mucoadhesive tests with anionic mucin dispersions. Blank liposomes and chitosomes showed no harmful effects on human laryngeal stromal cells; however, chitosomes effectively absorbed laryngeal cancer cells. Docetaxel-loaded chitosomes demonstrated much higher cytotoxicity against laryngeal cancer cells than stromal cells or control treatments. After 3 h of exposure, human red blood cells showed no hemolysis, indicating that intra-arterial injection is viable. These *in vitro* results indicated docetaxel-loaded chitosomes potential for locoregional chemotherapy delivery.^36^ Fig. 3. Schematic representation and experimental overview of docetaxel-loaded chitosomes designed for targeted intra-arterial delivery in laryngeal cancer therapy.

### 3.4 Iontophoresis-enhanced cisplatin delivery with chitosan nanoparticles

Iontophoresis has shown promise in improving cisplatin release from chitosan nanoparticles for oral cancer therapy. Cisplatin-encapsulated chitosan nanoparticles were created utilizing a variety of chitosan-to-TPP ratios, with the 15:1 ratio producing the maximum encapsulation efficiency (15.6 %) and allowing for continuous release for up to 35 days. Cellular uptake tests revealed that delivery happened by endocytosis. The MTT test demonstrated a dose-dependent reduction in cell viability. Cyclic chronopotentiometry (1 mA, on: off = 1s:1s) and differential pulse voltammetry (0.06 V) were the most efficient in tumor suppression, as demonstrated by reduced Ki-67 and pan CK expression. These findings indicate that iontophoresis is a potential strategy for targeted cisplatin administration in the treatment of oral cancer.^37^

### 3.5 Catechol-modified chitosan nanoparticles for doxorubicin delivery

Research examined the use of catechol-modified chitosan/hyaluronic acid nanoparticles (Cat-NPs) as carriers for doxorubicin (DOX) in oral cancer therapy. These nanoparticles bind well to the oral mucosa, allowing for long-term local medication administration. They are negatively charged, round, and about 160 nm in size. Cat-NPs exhibited higher mucoadhesion on porcine oral tissues than unmodified versions. The high DOX loading capacity of 250 μg/mg ensured regulated medication release. DOX-loaded Cat-NPs efficiently suppressed HN22 oral squamous carcinoma cells, with enhanced uptake, accumulation, and apoptosis induction as compared to free DOX. These data demonstrate Cat-NPs as a potential approach for localized oral cancer treatment.^38^

### 3.6 Epigallocatechin gallate (EGCG)-loaded alginate nanoparticles

The potential of epigallocatechin gallate (EGCG), a green tea-derived anticancer compound, encapsulated in sodium alginate nanoparticles (SA NPs), was investigated to enhance therapeutic efficacy. Five different SA NP sizes were synthesized, with EGCG incorporated into selected particles. Cytotoxicity tests showed that type 1 NPs at 80 μg/mL had the highest anticancer activity against TSCC-1 cancer cells. Further testing, including colony formation and wound healing studies, demonstrated a substantial reduction in tumor cell proliferation and migration. Apoptosis experiments demonstrated an increase in cancer cell death following treatment with EGCG-loaded NPs. These results indicate that EGCG-loaded SA NPs effectively limit cancer cell proliferation and migration while boosting apoptosis.^39^

### 3.7 Simvastatin-loaded chitosan-alginate nanoparticles for oral cancer

Rizg et al. (2022) developed and improved a chitosan-alginate nanoparticle system that encapsulates simvastatin (SIM-CA-NP) using a novel polyelectrolytic complexation process. For optimization, a central composite design was adopted, with chitosan and alginate concentrations changed over five levels to achieve the smallest particle size and best entrapment efficiency. The formulation of 13 trial runs was monitored by Design-Expert software, with an optimum composition of 0.258 g chitosan and 0.353 g alginate, yielding a particle size of 142.56 nm and an entrapment effectiveness of 75.18 %. The improved formulation (O-SIM-CAN) produced data that nearly matched theoretical expectations, therefore proving the design correctness. SIM release was maintained for more than 96 h, demonstrating successful encapsulation. Cell viability and Caspase-3 enzyme assays demonstrated enhanced anti-proliferative activity compared to free SIM and control groups in HCS-3 (human tongue squamous carcinoma cell line) cells. These findings suggested that the chitosan-alginate nanoparticle system improved the therapeutic potential of simvastatin.^40^

### 3.8 Chitosan nanoparticles against DMBA-induced hamster buccal pouch carcinogenesis

Ursolic acid, a natural pentacyclic triterpenoid, possesses strong anticancer properties but is limited by poor solubility and bioavailability. To overcome this, Karthik et al. (2023) developed ursolic acid-loaded chitosan nanoparticles (UACNPs) and evaluated their anticancer potential against DMBA-induced hamster buccal pouch carcinogenesis. In their studies, demonstrated that UACNP treatment significantly reduced tumor incidence, size, and burden. The treatment notably restored antioxidant enzyme activities (SOD, CAT, GPx) and decreased lipid peroxidation, indicating improved redox balance. Furthermore, immunohistochemical analysis revealed that UACNPs effectively modulated apoptotic signaling by downregulating anti-apoptotic markers (mutant p53 and Bcl-2) and upregulating pro-apoptotic proteins (Bax, Bid, Bad, caspase-3, and caspase-9), thus promoting apoptosis. These results highlight the dual action of UACNPs through antioxidant and pro-apoptotic mechanisms, supporting their potential as a nanotechnology-based chemopreventive and therapeutic agent for oral cancer.^41^^,^^42^

Mariadoss et al. reported that phloretin-loaded chitosan nanoparticles (PhCsNPs) exhibited a spherical morphology with a particle size of 80–100 nm and demonstrated stable, controlled drug release. In human oral cancer cells, PhCsNPs significantly induced cytotoxicity, elevated intracellular ROS levels, caused mitochondrial dysfunction, enhanced lipid peroxidation, and depleted antioxidant enzyme levels. They also triggered apoptosis through upregulation of mitochondrial-mediated apoptotic genes and induced cell cycle arrest, confirming their potential anticancer efficacy. Furthermore, *in vivo* studies using DMBA-induced OSCC in golden Syrian hamsters revealed that oral administration of PhCsNPs (5, 10, 20 mg/kg b.wt.) on alternate days for 14 weeks significantly attenuated tumor progression. This was evidenced by reduced lipid peroxidation and restoration of antioxidant and detoxification enzymes in plasma, erythrocytes, buccal, and liver tissues, as well as histopathological tumor regression. Western blot analysis confirmed mitochondrial-mediated apoptosis through upregulation of Bax, cytochrome *c*, caspase-3, and caspase-9, and downregulation of Bcl-2. These findings highlight the potent antioxidant and anticancer potential of PhCsNPs.^43^^,^^44^

## 4 Cyclodextrin-based nanoformulations for HNS treatments

Cyclodextrins are cyclic oligosaccharides composed of glucose units linked by α-1,4-glycosidic bonds. They form a truncated cone-shaped structure with a hydrophobic interior and a hydrophilic outer surface. This architecture enables them to form inclusion complexes with a variety of molecules, thereby improving solubility and stability.^45^ Due to their biocompatibility and ability to encapsulate poorly soluble drugs, cyclodextrins are widely used in nanoparticle-based pharmaceutical delivery.^46^ Typically derived from starch via enzymatic conversion using cyclodextrin glucanotransferase (CGTase), the most common forms: α-, β-, and γ-cyclodextrins, vary in glucose unit number.^47^ Their application in drug nanocarriers allows for targeted and controlled release, thereby enhancing therapeutic efficacy.^48^

### 4.1 β-Cyclodextrin nanosponges in photodynamic therapy

PDT works by activating a photosensitizer with light to kill malignant or precancerous cells. Although photosensitizer uptake has traditionally been emphasized, drug penetration has received less attention. Recent studies reveal that β-cyclodextrin polymer (β-CDp) nanosponges can improve drug penetration while reducing cellular absorption in multicellular HNC spheroids. When delivering temoporfin to stroma-rich HNC models, these nanosponges allowed deeper and more uniform distribution, despite cutting cellular uptake by 50 % compared to free drug. Remarkably, PDT efficacy remained comparable, suggesting that β-CDp nanosponges may enhance *in vivo* performance by limiting off-target toxicity without sacrificing therapeutic outcomes.^49^

### 4.2 HP-β-CD and lidocaine: enhanced cytotoxicity in SCC cells

A study by Ferreira et al. (2018) investigated the cytotoxic effects of lidocaine (lido) and its HP-β-cyclodextrin (HP-β-CD) complex on SCC9 and SCC25 head and neck squamous carcinoma cell lines. The HP-β-CD–lido inclusion complex significantly reduced cell viability to 63 % in SCC9 and 44 % in SCC25, compared to lido alone (83 % and 71 %, respectively). Cell proliferation assays also confirmed that HP-β-CD complexation enhanced lidocaine's cytotoxic effects.^50^ This underscores HP-β-CD's role in potentiating therapeutic efficacy against HNC cells.

### 4.3 Cyclodextrin-based buccal delivery of paclitaxel

A buccal delivery system for paclitaxel (PTX) was developed using Pluronic F127 (PF127) and polyethylene oxide (PEO), aiming to improve solubility and mucosal adhesion. PTX was combined with DMβCD to form an inclusion complex and incorporated into hydrogels of varying polymer concentrations. Using a Franz diffusion cell in phosphate-buffered saline at 37 °C, drug release patterns were evaluated. The mucoadhesive polymer PEO slowed release, while PF127 influenced the sol-gel transition temperature. Cytotoxicity was validated through MTT assays on KB cells, showing potent effects. This delivery platform holds promise for local chemotherapy via the oral mucosa.^51^

### 4.4 HPβCD-resveratrol formulations for OSCC chemoprevention

Resveratrol (RV) is under investigation for its chemopreventive potential against OSCC originating from oral preneoplastic lesions (OPLs). To improve bioavailability, RV was complexed with 2-hydroxypropyl-β-cyclodextrin (HPβCD), overcoming its rapid metabolic degradation. *In vitro* tests on HCPC I cells and an *in vivo* DMBA-induced hamster model revealed that both RV and RV–HPβCD significantly reduced OPL and OSCC formation. The mouthwash formulation of RV–HPβCD showed superior efficacy, with HPLC confirming enhanced concentration-dependent activity.^52^ These findings support RV–HPβCD as a viable chemopreventive strategy in early oral cancer management.

## 5 Poly (lactic acid), poly (caprolactone) and poly (ethylene glycol) nanoparticles on HNC

PLA, PCL, and PEG nanoparticles are biodegradable and biocompatible polymers with several uses in drug delivery and biomedicine.^53^ PLA is obtained from renewable resources such as maize starch or sugarcane and has a linear aliphatic polyester structure made up of repeating lactic acid units.^54^ PCL is made via ring-opening polymerization of ε-caprolactone. It has a semi-crystalline structure and modest degradation rates, making it ideal for long-term use.^55^ PEG is a hydrophilic polyether obtained through the polymerization of ethylene oxide and is often used to improve nanoparticle solubility and circulation time in the body.^56^ When combined, PLA or PCL provides mechanical strength and biodegradability, while PEG imparts stealth properties that reduce immune recognition.^57^ These polymers can be blended or chemically linked to form copolymers like PLA-PEG or PCL-PEG. Such copolymers self-assemble into nanoparticles with core-shell structures, where the hydrophobic core encapsulates drugs and the hydrophilic shell stabilizes the particle in aqueous environments. The tunable properties of these polymers enable controlled drug release and targeted delivery.^58^ PLA nanoparticles sustain release over days to weeks, degrading hydrolytically to lactic acid metabolized to CO_2_ and water. PCL degrades slowly, allowing prolonged pharmacokinetic profiles, with uptake by endocytosis and clearance as hydrolytic 6-hydroxycaproic acid. PEG coating (PEGylation) extends circulation (200–1000 min), reducing immune clearance and renally excreted after detachment. PLA, PCL, and PEG are approved by major regulatory authorities such as the U.S. Food and Drug Administration (FDA) and the European Medicines Agency (EMA), which enhances their relevance for clinical applications.^59^

### 5.1 PLA-NPs loaded with CuB and CASC2c for oral cancer treatment

PLA possessed nanoparticle characteristics and functioned as effective drug carriers. Wang and Zhang (2021) conducted a study utilizing PLA-NPs to develop nano-drugs incorporating cucurbitacin B (CuB) and long non-coding RNA CASC2c to assess their impact on oral cancer cell progression. CuB-PLA-NPs and CuB-PLA-NPs-CASC2c were synthesized, followed by an analysis of their properties and drug release behavior. Results indicated that CuB-PLA-NPs exhibited 90 % drug release within 24 h, whereas CuB-PLA-NPs-CASC2c displayed a sustained release profile (62 %) over 72 h. The CuB-PLA-NPs-CASC2c treatment significantly increased apoptosis (90 %) and inhibited migration and invasion. Additionally, CASC2c upregulation corresponded with decreased STAT3, MMP-2, and VEGF levels, suppressing angiogenesis and oral cancer progression.^60^

### 5.2 CDDP/CQ-PLA nanoparticles induce apoptosis and oxidative stress in OSCC

A study investigated the effects of PLA-based nanoparticles loaded with cisplatin and chloroquine (CDDP/CQ-PLA NPs) on OSCC Cal-27 cells. Cytotoxicity assays, including MTT and colony formation tests, demonstrated that CDDP/CQ-PLA NPs exhibited stronger cytotoxicity compared to CDDP-PLA NPs and free cisplatin. Apoptosis analysis using Annexin/PI staining and western blotting revealed that CDDP/CQ-PLA NPs triggered caspase-dependent apoptosis. Further investigation revealed that these nanoparticles caused oxidative stress by raising reactive oxygen species (ROS) generation and malondialdehyde (MDA) levels, while lowering antioxidant enzyme activity. Furthermore, CDDP/CQ-PLA NPs hindered autophagy, as evidenced by lower LC3-II levels and higher p62 protein expression. These findings imply that CDDP/CQ-PLA NPs improve cisplatin's anticancer activity by inducing apoptosis and oxidative damage in OSCC cells while reducing autophagy.^61^

### 5.3 PDA@CUR@PCL/PLA injectable fiber system for synergistic OSCC therapy

Chen et al. (2025) developed an injectable fiber system, polydopamine@curcumin@polycaprolactone/polylactic acid (PDA@CUR@PCL/PLA), designed to address the limitations of conventional implantable fibrous membranes for the treatment of OSCC. These short fibers are loaded with curcumin (CUR) and coated with polydopamine (PDA), which enhances biocompatibility and imparts robust photothermal properties. Upon exposure to near-infrared (NIR) light, the PDA coating facilitates localized heat generation, effectively inducing damage to cancer cells. Concurrently, CUR is released in a pH-responsive manner, targeting the acidic tumor microenvironment and providing sustained anticancer activity. Both *in vitro* and *in vivo* evaluations demonstrated that this dual-functional fiber system significantly inhibited OSCC cell proliferation through a synergistic combination of photothermal therapy and chemotherapy, with minimal toxicity to surrounding healthy tissues. This injectable fiber-based approach offers a promising, minimally invasive strategy for effective OSCC therapy.^62^

### 5.4 DOX-loaded mPEG-PLA-OA micelles enhancing oral cancer therapy

An amphiphilic polymer, mPEG-PLA-OA (OA: Oleanolic acid), was developed to self-assemble in an aqueous environment and effectively encapsulate DOX. *In vitro* studies using the oral cancer cell line FaDu (HTB-43) demonstrated enhanced cytotoxicity, increased apoptosis, and significant mitochondrial disruption compared to free DOX. Experiments with 3D cultures further confirmed deeper tumor penetration and stronger inhibitory effects. Pharmacokinetic analysis revealed prolonged circulation time and reduced clearance, without significant organ toxicity. These results underscore the potential of DOX-loaded mPEG-PLA-OA micelles as a promising alternative for oral cancer therapy.^63^

### 5.5 PLGA nanoparticles with ultra-high PTX loading via QbD strategy

Using a quality-by-design (QbD) strategy, researchers created ultra-high PTX-loaded poly (lactide-co-glycolide) nanoparticles. The improved formulation yielded ultra-small spherical particles (∼53 nm) with good encapsulation effectiveness (>90 %) and low polydispersity index (0.221). It increased *in vitro* drug release by tenfold over 72 h when compared to free PTX. In FaDu cells (human pharyngeal squamous cell carcinoma), the nanoparticles reduced cell viability by 50 % within 24 h, significantly outperforming free PTX. They also enhanced intracellular uptake and improved PTX antitumor activity by reducing its IC50 by nearly 50 %. These results underscore the potential of quality by design-optimized PLGA nanoparticles for improved drug delivery and therapeutic efficacy.^64^

### 5.6 Dual-drug delivery via HA/CS-coated PLGA nanoparticles for oral cancer

Mesrati et al. (2023) studied and analyzed hyaluronic acid/chitosan-coated poly (lactic-co-glycolic acid) nanoparticles (HA/CS-coated PLGA NPs) for the delivery of PTX and Temozolomide to human tongue squamous cell carcinoma cells with high CD44 expression. The study examined how these medications may be administered together to see whether they had any synergistic benefits while keeping drug concentration to a minimum. The nanoparticles were synthesized using solvent evaporation and measured 260.40 ± 11.54 nm. They had a positive zeta potential of +14.31 ± 1.37 mV and a low polydispersity index of 0.15 ± 0.03. Drug-loaded nanoparticles showed better cell inhibition than free medicines, with lower half-maximal inhibitory doses. Enhanced apoptotic activity, S-phase arrest, increased ROS production, mitochondrial dysfunction, and upregulated genes related to cell inhibition and death were observed in CAL-27 cells. These findings highlighted the potential of the developed nanoparticles in improving the efficacy of single and dual drug therapies for oral cancer treatment.^65^

### 5.7 Photodynamic therapy using ruthenium (II)-loaded PLGA nanoparticles

PDT was investigated using a ruthenium (II) polypyridyl complex as a photosensitizer. When exposed to blue-green light, this novel Ru(II) complex containing 2,2′-biimidazole and tetramethylphenanthroline ligands generated both superoxide anion and singlet oxygen. To promote solubility and bioavailability, the complex was encapsulated in self-assembled hyaluronic acid-poly (lactic-co-glycolic acid) nanoparticles, resulting in controlled release and higher stability. The nanoparticles had a high encapsulation efficiency (70 %), a low polydispersity index (0.12), and accelerated release in cancer cells due to hyaluronidase enzyme activity. Cellular absorption tests in TR146 oral cancer cells indicated significant internalization, resulting in more than 90 % cytotoxicity when activated with 470 nm light. These findings demonstrate the Ru(II) complex-loaded nanocarrier's potential as an effective PDT agent for treating oral cancer.^66^

### 5.8 Lipid-coated nanoparticles of curcumin for HPV-positive and negative HNSCC

**Fig. 4:**
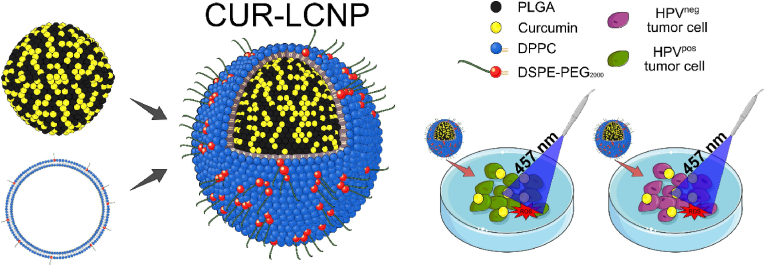
Schematic illustration of CUR-LCNP structure comprising PLGA, curcumin, DPPC, and DSPE-PEG_2000_, and its selective phototoxic effect on HPV^+^ versus HPV^−^ tumor cells under 457 nm light via ROS generation^67^.

CUR-LCNPs, lipid-coated polymeric nanoparticles containing curcumin, have been developed as a possible PDT therapy for HNSCC. The nanoparticles had a size of 153.37 ± 1.58 nm and an encapsulation effectiveness of 92.69 ± 0.03 %. HPV-positive (UM-SCC-47, UPCI-SCC-154, and UM-SCC-104) and HPV-negative (UM-SCC-3, UM-SCC-27, and UT-SCC-26A) HNSCC cell lines showed significant tumor cell inhibition, with no significant differences between groups (IC_50_: 9.34 ± 4.73 μmol/L vs. 6.88 ± 1.03 μmol/L). The nanoparticles were created using nanoprecipitation with PLGA, followed by lipid coating utilizing a fusion technique. The findings identified CUR-LCNPs as a viable therapy option for HNSCC, regardless of HPV status (Fig. 4).^67^

### 5.9 mTHPP-loaded micelles for targeted photodynamic therapy

Cohen et al. (2010) investigated the encapsulation of 5,10,15,20-tetrakis (meso-hydroxyphenyl)porphyrin (mTHPP) into polymeric micelles (made of PEG and PLA) to increase its solubility and efficacy in PDT against HNC cells. The micelles, which were created and studied using ultraviolet–visible and fluorescence spectroscopy, had a constant size of about 30 nm. Transmission electron microscopy and dynamic light scattering confirmed the findings. Encapsulation efficiency reached 85 % at loading levels of up to 17 %. The study looked at the efficiency of PDT on HSC-3 and HN-5 cancer cells. Laser irradiation at 420 nm caused over 90 % cytotoxicity, whereas dark toxicity was less than 10 % at a dose of 25 μg/mL. These findings suggested that mTHPP-loaded micelles might be a promising approach to targeted cancer treatment.^68^

### 5.10 Paclitaxel-loaded polymeric nanoparticles based on α-tocopheryl succinate for the treatment of HNSCC

The study by Juan Riestra-Ayora et al. (2021) explores a novel approach to treating HNSCC using paclitaxel-loaded polymeric nanoparticles based on α-tocopheryl succinate (α-TOS). Recognizing the limitations and side effects associated with conventional paclitaxel (PTX) therapy, the authors developed a nanocarrier system composed of block copolymers of polyethylene glycol (PEG) and a methacrylic derivative of α-TOS. These nanoparticles were administered via direct intratumoral injection in a murine xenograft model using FaDu hypopharyngeal squamous carcinoma cells. The study demonstrated that PTX-loaded nanoparticles (PTX-NPs) significantly enhanced antitumor efficacy compared to free PTX, evidenced by reduced tumor volume, increased apoptosis, and elevated oxidative and nitrosative stress within tumor tissues. Furthermore, immunohistochemical analysis revealed a marked decrease in proliferation (Ki-67), angiogenesis (CD31, CD34, Factor VIII), and EGFR expression in the PTX-NP-treated group. The use of α-TOS not only served as a biodegradable polymer but also contributed therapeutic pro-apoptotic effects, making the system dual-functional.^69^

### 5.11 Targeted peptide-modified nanoparticles for irinotecan and miR-200 delivery

A recent study found that liposomes and solid lipid nanoparticles (SLNs) treated with a self-destructive PEG shell and different targeting peptides improved the distribution of irinotecan and miR-200. These peptide-modified nanoparticles were engineered to enhance tumor specificity and cellular uptake. The co-treatment significantly induced apoptosis in SAS cells by modulating key signaling pathways, ultimately outperforming standard treatments and presenting a novel, effective strategy for HNC therapy.^70^

### 5.12 Folate-targeted nanoparticles for honokiol delivery in nasopharyngeal cancer

Building on the possibility of targeted drug delivery, another study created a mechanism for the persistent and selective release of honokiol (HK) to nasopharyngeal cancer (NPC) HNE-1 cells by leveraging folate receptor overexpression. Active targeting nanoparticles loaded with HK (ATNH) were manufactured by emulsion solvent evaporation, which included a folate-modified PCEC copolymer and polyethyleneimine. *In vitro* evaluations, including MTT assay, cellular uptake, and apoptosis analysis, demonstrated strong antitumor effects. Complementary *in vivo* studies such as cell-cycle analysis, PET/CT imaging, and immunohistochemistry further confirmed tumor growth inhibition, metabolic reduction, and cell-cycle arrest. These findings highlight ATNH as a promising therapeutic approach for NPC treatment.^71^

### 5.13 PCL nanoparticles for combined cisplatin and curcumin delivery

Pornpitchanarong et al. (2021) studied the effects of cisplatin and curcumin-encapsulated polycaprolactone nanoparticles (PCL-NPs) on oral epidermal carcinoma cells. The nanoparticles were generated using the nanoprecipitation method, which included poly (vinyl alcohol) and polysorbate 80 as stabilizers. The synthesized nanoparticles were smaller than 300 nm and had a limited distribution, resulting in excellent drug loading efficiencies. The combination of cisplatin and curcumin in the delivery system showed higher anticancer activity, underlining PCL-NPs' potential for treating oral cancer.^72^

### 5.14 Targeting PI3K/AKT pathway in HNSCC using NP-427

Recent studies into HNSCC have shown critical molecular pathways involved in tumor formation. Among them, the phosphatidylinositol-3-kinase (PI3K) signaling pathway has emerged as an important therapeutic target. PHT-427, a PI3K and AKT/PDK1 dual inhibitor, was investigated for anticancer effectiveness when encapsulated in polymeric nanoparticles (NP-427) and given intratumorally in a hypopharyngeal squamous cell carcinoma xenograft mouse model. The nanocarrier system, made of block copolymers of N-vinylpyrrolidone and a methacrylic derivative of α-TOS, was designed for efficient drug delivery. NP-427 therapy improved efficacy by decreasing tumor volume, decreasing PI3K/AKT/PDK1 expression, and boosting tumor necrosis. Furthermore, NP-427-treated tumors showed decreased levels of Epidermal Growth Factor Receptor (EGFR) and angiogenesis indicators, indicating better treatment effects.^73^

### 5.15 Chemoprevention with polydatin-loaded PLGA nanoparticles

Vijayalakshmi et al. (2019) reported that polydatin-loaded PLGA nanoparticles (POL-PLGA-NPs) significantly reduced tumor incidence and burden in DMBA-induced oral carcinogenesis. The treatment restored antioxidant enzymes (SOD, CAT, GSH), enhanced detoxifying enzymes (cytochrome P450, GST), and normalized histopathological features. Additionally, POL-PLGA-NPs induced apoptosis via upregulation of p53, Bax, cleaved caspase-3, and downregulation of Bcl-2, indicating strong chemopreventive potential.^74^

### 5.16 Nedaplatin-loaded PLGA nanoparticles target notch signaling in OSCC

Senkuttuvan Ilanchit Chenni et al. (2024) demonstrated the tumor-targeting potential of PLGA-loaded Nedaplatin (PLGA-NDP) nanoparticles in a 7,12-dimethylbenz [a]anthracene (DMBA)-induced OSCC model using Syrian hamsters. PLGA-NDP exhibited marked antitumor efficacy, as evidenced by significant suppression of tumor formation and histological features associated with well-differentiated OSCC. Notably, PLGA-NDP downregulated oncogenic and anti-apoptotic markers such as Bcl-2, Bcl-xL, p21, PGE2, HGF, and CXCL12, while upregulating pro-apoptotic proteins including p53 and Bax, highlighting its ability to induce apoptosis. Moreover, RT-PCR and immunoblotting analyses confirmed the downregulation of key genes in the Notch signaling pathway (Notch1, Notch2, Hes1, Hey1, Jagged1), suggesting that PLGA-NDP-mediated tumor suppression is partly due to the inhibition of Notch signaling. These findings underscore the therapeutic potential of PLGA-based nanoparticles for oral cancer treatment. ^75^

### 5.17 Overcoming chemoresistance with proanthocyanidin-loaded CS-PLGA nanoparticles

The study by Yuan et al. (2024) demonstrates that chitosan-PLGA-based nanoparticles encapsulating Procyanidin (CS-PLGA-PHL) represent a promising therapeutic strategy for overcoming chemoresistance in OSCC. The nanoparticles exhibited favorable physicochemical properties and effectively suppressed the proliferation, migration, and invasion of drug-resistant OSCC cells. Mechanistically, the formulation downregulated MMP-2 and MMP-9 expression, highlighting its role in impairing metastatic potential.^76^

### 5.18 TSN-PLGA nanoparticles enhance apoptosis in OSCC

Chen et al. (2025) developed a poly (lactic-co-glycolic acid)-based nanoparticle formulation encapsulating Toosendanin (TSN) to overcome its limitations of poor water solubility and bioavailability. The engineered TSN-PLGA nanoparticles exhibited a sustained *in vitro* release profile, improved physicochemical characteristics, and demonstrated potent cytotoxicity against OSCC cells, inducing apoptosis and cell-cycle arrest in the S-phase at low doses. Mechanistically, RNA-sequencing analysis identified the involvement of JAK/STAT and PI3K–Akt signaling pathways in mediating the antitumor effects. Notably, *in vivo* studies in nude mice confirmed the antitumor efficacy and biosafety of TSN-PLGA NPs without observable systemic toxicity.^77^

### 5.19 Paclitaxel-loaded PLGA nanoparticles enhance drug delivery in pharyngeal carcinoma cells

Haider et al. (2020) successfully optimized paclitaxel-loaded PLGA nanoparticles (PTX-PLGA-NPs) using a quality-by-design (QbD) approach to enhance drug delivery in head and neck cancer treatment. Compared to free paclitaxel, the optimized formulation demonstrated a 10-fold increase *in vitro* drug release over 72 h and significantly improved cytotoxic effects, with a nearly 50 % reduction in cell viability and IC_50_ values against pharyngeal carcinoma cells. Furthermore, the nanoparticles showed enhanced intracellular uptake, attributed to their nano-scale size and negative surface charge (−10.1 mV).^78^

### 5.20 PEG-PCL micellar encapsulation of icaritin for targeted OSCC therapy

Yang et al. (2021) developed a nanocarrier system using amphiphilic poly (ethylene glycol)-poly (ε-caprolactone) (PEG-PCL) micelles for the effective delivery of icaritin, a bioactive compound known to exert anti-proliferative and pro-apoptotic effects in OSCC. Due to the poor water solubility and bioavailability of free icaritin, its clinical application has been limited. To overcome this, icaritin was encapsulated within PEG-PCL micelles. The nanoformulation demonstrated enhanced colloidal stability and a sustained-release profile, enabling prolonged drug exposure to cancer cells.^79^

### 5.21 PLGA-based dexamethasone nanoparticles for mitigating oral mucositis

Ribeiro et al. (2021) reported the development of dexamethasone-loaded poly (lactic-co-glycolic acid) (PLGA) nanoparticles as a promising therapeutic approach for 5-fluorouracil (5-FU) induced oral mucositis, one of the most debilitating side effects in HNC patients undergoing chemoradiotherapy. The PLGA nanocarrier system was synthesized using an emulsification and solvent evaporation technique and demonstrated favorable physicochemical characteristics. *In vivo* studies using a hamster model showed that PLGA-DEX nanoparticles significantly reduced oral mucosal inflammation, suppressed TNF-α and IL-1β, reduced oxidative stress, and modulated gene expression of NF-κB, COX-2, TGF-β, GILZ and MKP1.^80^

## 6 Dendrimers and micelles nanoparticles on HNC

Dendrimers are highly branching, tree-like polymers having a central core and layers (generations) of repeating units, resulting in a well-defined and symmetrical structure.^81^ Micelles are spherical structures generated by the self-assembly of amphiphilic molecules in aqueous solutions, having hydrophobic cores and hydrophilic shells.^82^ Dendrimers are synthetically generated with exact molecular weight and functionality, whereas micelles are often made from surfactants or block copolymers.^81^^,^^82^ Dendrimers exhibit size and generation dependent pharmacokinetic, internalized via endocytosis, with drug release through bond cleavage and renal or RES elimination. Polymeric micelles (PCL–dextran, PEG copolymers) avoid renal clearance, enter via endocytosis, release drugs in response to stimuli, and degrade hydrolytically or enzymatically. Both structures can encapsulate drugs, dendrimers via internal cavities and surface binding and micelles via their hydrophobic core. These nanocarriers enhance solubility, stability and targeted delivery of therapeutic agents.

### 6.1 Folic acid-functionalized dendrimers for targeted gene and siRNA delivery in HNC

A recent study revealed the substantial potential of folic acid (FA)-decorated polyamidoamine dendrimer G4 (G4-FA) as a targeted gene and siRNA delivery platform for HNC treatment. G4-FA was successful in targeting cancer cells that overexpress folate receptors (FRs), competing with free FA for the same binding site, promoting FR-dependent absorption of DNA plasmids, and selectively increasing gene expression in FR-high cancer cells. These findings demonstrated its targeting specificity and cytocompatibility, which support its usage as a gene delivery vector. Furthermore, in a xenograft HN12 tumor mouse model of HNSCC, intratumoral treatment of G4-FA resulted in increased tumor uptake and longer retention, as shown by near infrared (NIR) imaging. When complexed with therapeutic siRNA targeting VEGFA (siVEGFA), G4-FA led to significant tumor growth inhibition compared to controls. A two-dose regimen administered eight days apart achieved greater suppression of tumor progression and angiogenesis, confirmed by reduced CD31 staining, lower microvessel counts, and fluorescence imaging. Collectively, these findings highlight G4-FA's promise as an effective and localized delivery system for gene and siRNA-based cancer therapies.^83^^,^^84^

### 6.2 Folate receptor-targeted chemotherapy using dendrimer-methotrexate conjugates

A study on HNSCC utilized a heterotopic tumor model to evaluate the importance of targeting the folate receptor. It was found that the expression levels of folate-binding protein alpha (FBP-α) significantly influenced treatment efficacy. Dendrimers conjugated with folic acid and methotrexate were synthesized and administered to SCID mice bearing various UM-SCC tumor lines. Targeted therapy demonstrated superior outcomes compared to free methotrexate or saline, particularly in tumors with high FBP-α expression. Moreover, this approach allowed for administration of drug doses three times higher than the free form, with reduced systemic toxicity. Real-time PCR was used to quantify FBP-α expression, informing personalized treatment strategies. These findings suggest that dendrimer-based chemotherapy can be effective even in tumors with lower receptor expression than those typically studied.^85^

### 6.3 Dual-modality imaging using polymeric nanomicelles in HNC

Another study found that polymeric phospholipid-based nanomicelles have the potential for dual-modality imaging of HNC. Nanoparticles encapsulated in Pt (TPNP) and functionalized with gadolinium were produced and studied for optical and magnetic characteristics. These nanomicelles, which are around 100 nm in size, remained persistent in aquatic settings and showed significant tumor localization. MRI data revealed increased contrast near the tumor's perimeter, which remained for up to 24 h after injection. Near-infrared (NIR) imaging further confirmed nanoparticle accumulation within tumors, aided by minimal background interference due to the wide spectral separation. This dual-imaging technique provided both anatomical and functional insights into tumor biology, laying the groundwork for future applications in imaging and therapy.^86^

### 6.4 Curcumin-loaded nanomicelles to overcome multidrug resistance in oral cancer

CUR, despite its proven anticancer effects, has poor water solubility and bioavailability, limiting its usefulness. Kumbar et al. (2022) produced curcumin nanomicelles (CUR-NMs) utilizing DSPE-PEG-2000 to improve curcumin delivery. CUR-NMs showed increased cellular absorption and cytotoxicity against cisplatin-resistant KB cancer cells. These findings showed that CUR-NMs might be a potential alternative treatment option for multidrug-resistant oral cancer.^87^

### 6.5 Polymeric micelles for enhanced photodynamic therapy using mTHPP

The encapsulation of 5,10,15,20-tetrakis (meso-hydroxyphenyl)porphyrin (mTHPP) into polymeric micelles has shown promising outcomes for PDT. *In vitro* experiments using HSC-3 and HN-5 human HNC cells revealed substantial cytotoxicity upon light activation. Confocal microscopy and MTT assay results confirmed therapeutic efficacy, showing over 90 % phototoxicity with minimal dark toxicity. These findings underscore the significance of polymeric micelles as effective nanocarriers for hydrophobic photosensitizers in targeted cancer treatment.^88^

Further investigation into the role of varying mTHPP loading percentages revealed a notable impact on fluorescence intensity, singlet oxygen (^1^O_2_) generation, and cytotoxicity in HN5 and H2009 cancer cell lines. Micelles loaded with 2 % mTHPP exhibited the highest ^1^O_2_ yield and phototoxicity while maintaining minimal dark toxicity, indicating an optimal therapeutic effect. Interestingly, a 0.5 % loading produced the strongest fluorescence signal in solution, whereas higher loadings resulted in enhanced intracellular fluorescence. Cytotoxic responses in both cell lines reinforced the micelles' effectiveness under light activation. These insights contribute to a deeper understanding of drug-loading effects on micellar performance and support the development of safer, more effective PDT formulations targeting HNCs.^89^

### 6.6 Dendrimer-based RNAi therapy targeting hTERT in oral cancer

Liu et al. (2011) demonstrated that polyamidoamine (PAMAM) dendrimer-delivered short hairpin RNA (shRNA) targeting human telomerase reverse transcriptase (hTERT) effectively suppressed oral cancer cell growth *in vitro* and *in vivo*. The dendrimer-shRNA complex successfully silenced the hTERT gene, leading to significant inhibition of cell proliferation and induction of apoptosis. Furthermore, in a xenograft mouse model, treatment with this shRNA-dendriplex resulted in marked tumor growth reduction. This study highlights the potential of dendrimer-based RNAi therapy targeting hTERT as a novel and efficient strategy for oral cancer treatment.^90^

### 6.7 Oleanolic acid-functionalized micelles for doxorubicin delivery in oral cancer

Kumbham et al. developed oleanolic acid (OA)-conjugated poly (D, L-lactide)-based polymeric micelles (mPEG-PLA-OA) for the co-delivery of doxorubicin (DOX) in oral cancer therapy. These micelles demonstrated efficient drug loading, sustained release, and enhanced physicochemical stability. The incorporation of OA not only facilitated active targeting toward tumor cells but also contributed to synergistic anticancer effects due to its intrinsic therapeutic properties. *In vitro* studies showed significantly higher cellular uptake, enhanced cytotoxicity, and increased apoptosis in oral cancer cells when treated with DOX-loaded micelles compared to free DOX. Furthermore, 3D tumor spheroid models confirmed superior penetration and antitumor activity. *In vivo* pharmacokinetic studies revealed prolonged systemic circulation and reduced cardiotoxicity. These findings suggest that the OA-functionalized micellar nanocarrier significantly improves the therapeutic efficacy and safety of DOX and holds promise as a targeted delivery platform for oral cancer treatment.^91^

### 6.8 CD44-targeted micelles (mP6/Rg3) induce ferroptosis in oral cancer stem cells

Cai et al. (2025) demonstrated that CD44-targeted micelles (mP6/Rg3), composed of the CD44-binding peptide P6 and ginsenoside Rg3, exerted potent therapeutic effects against oral cancer stem cells (CSCs). The micelles significantly inhibited the expression of ABCB1 (ATP-binding cassette subfamily B member 1), a key efflux transporter implicated in drug resistance and ferroptosis evasion. This suppression led to downstream downregulation of GPX4 (Glutathione Peroxidase 4), an essential enzyme that protects cells from lipid peroxidation, and altered the expression of ferroptosis-related genes, including SLC7A11 (a cystine/glutamate antiporter involved in glutathione synthesis) and FTH1 (ferritin heavy chain 1, which regulates iron storage). These molecular changes disrupted redox homeostasis and iron metabolism within CSCs, resulting in increased lipid peroxidation and induction of ferroptotic cell death. Furthermore, the treatment reduced the expression of stemness-associated markers such as CD44, ALDH1A1, and SOX2, indicating a loss of CSC characteristics. *In vivo* studies in OSCC-bearing models revealed marked tumor growth inhibition and a decrease in CSC frequency following mP6/Rg3 treatment. These findings collectively highlight that dual targeting of CD44 and ABCB1 using the mP6/Rg3 nanoplatform promotes ferroptosis and impairs cancer stemness, offering a promising therapeutic strategy for OSCC.^92^

### 6.9 Paclitaxel-loaded PEG-ODA micelles for targeted OSCC therapy

Yang et al. (2025) developed amphiphilic PEG-octadecylamine (PEG-ODA) block copolymer micelles encapsulated with paclitaxel (PTX) to overcome the limitations of PTX's low water solubility and enhance its tumor-targeting potential for OSCC treatment. The micelles displayed excellent colloidal stability over 24 h. *In vitro* studies using CAL-27 oral cancer cells demonstrated that the micelles were effectively internalized and significantly inhibited cell proliferation, as evidenced by CCK-8 assays and live/dead cell staining. *In vivo*, PEG-ODA-PTX-treated mice bearing CAL-27 xenograft tumors exhibited a tumor inhibition rate of 72 % and a notable reduction in tumor volume compared to controls (P < 0.05). Importantly, biosafety evaluations, including hemolysis testing, cytotoxicity assays, and histopathological examination of major organs via H&E staining, revealed no significant systemic toxicity. Overall, Yang et al. concluded that PEG-ODA-PTX micelles represent a promising and biocompatible nanocarrier platform for the effective and targeted delivery of paclitaxel in OSCC therapy.^93^

Table 1 provides a comparative overview of various polymeric nanoparticles investigated against HNC treatment, detailing their formulation types, loaded therapeutic agents, observed therapeutic outcomes, as well as their respective advantages and limitations.

**Table 1:** Comparative overview of various polymeric nanoparticles on head and neck cancer.

Nanoparticle	Drug	*In Vitro*/*In Vivo* Study	Findings	Advantages	Limitations	Ref.
CS-PCL Microparticles	5-Fluorouracil	CAL27, HSC3 cell lines; mouse model (AT84)	Sustained release (96 h), selective to cancer cells, induced apoptosis	Biocompatibility, prolonged release, tumor specificity	Limited penetration in deep tumor tissues	^34^
optEr/Cm-NP in CS/β-GP Hydrogel	Erlotinib + Curcumin	FaDu HNSCC cell line; Xenograft model	Enhanced uptake, significant tumor suppression	Dual-drug synergy, hydrogel-assisted delivery	Needs localized administration	^35^
Docetaxel-loaded Chitosomes	Docetaxel	Laryngeal cancer cells	High cytotoxicity, mucoadhesion, no hemolysis	Safe, site-specific intra-arterial potential	Only *in vitro* tested; long-term toxicity unknown	^36^
CS NPs + Iontophoresis	Cisplatin	Oral cancer cell line	Sustained release (35 days), improved uptake	Non-invasive enhancement, targeted delivery	Requires external device for iontophoresis	^37^
Cat-NPs (CS/HA)	Doxorubicin	HN22 cell line	Enhanced apoptosis, mucoadhesion	High loading, mucoadhesive for oral mucosa	Poor systemic bioavailability	^38^
SA NPs	EGCG	Human tongue squamous cell carcinoma (TSCC) cell line	Induced apoptosis, reduced migration	Natural antioxidant use, safer profile	Sensitive to degradation, poor solubility	^39^
SIM-CA-NP	Simvastatin	HCS-3 cell line	Enhanced apoptosis, optimized formulation	Repurposed drug, good bioactivity	Lipophilic nature may hinder delivery	^40^
Chitosan nanoparticles	Ursolic acid	DMBA-induced hamster buccal pouch carcinogenesis model	UACNPs significantly reduced tumor incidence, size, and burden; restored antioxidant enzymes; decreased lipid peroxidation; downregulated mutant p53 and Bcl-2; and upregulated Bax, Bid, Bad, caspase-3, and caspase-9.	Enhanced solubility and bioavailability of ursolic acid Dual mechanism: antioxidant and pro-apoptotic Effective chemoprevention in oral cancer	Requires further validation in human models Long-term safety and pharmacokinetics not assessed	^41^^,^^42^
Chitosan nanoparticles (PhCsNPs)	Phloretin	Human oral cancer cells and DMBA-induced OSCC in golden Syrian hamsters	Induced ROS, mitochondrial dysfunction, lipid peroxidation, apoptosis, and cell cycle arrest *in vitro.In vivo*, PhCsNPs reduced tumor progression, restored antioxidant and detox enzymes, and promoted mitochondrial-mediated apoptosis	Controlled and stable drug release; Potent antioxidant and pro-apoptotic effects; Effective oral cancer inhibition *in vivo*	No human clinical validation; Long-term safety data lacking	^43^^,^^44^
β-CDp Nanosponges	Temoporfin	Head and neck cancer spheroid model	Improved penetration, PDT efficacy	Effective in 3D tumor models	Low cellular uptake	^49^
HP-β-CD	Lidocaine	SCC9, SCC25 cancer cell lines	Greater cytotoxicity vs. free drug	Increased solubility and efficacy	Application limited to local treatment	^50^
DMβCD Hydrogel	Paclitaxel	KB cells	Improved solubility, effective cytotoxicity	Buccal delivery suitable	May not be ideal for systemic HNC	^51^
HPβCD	Resveratrol	HCPC I cells and an DMBA-induced hamster oral cancer model	Enhanced chemoprevention	Natural compound use, safe	Requires frequent dosing	^52^
PLA-NPs	CuB, CASC2c	Oral Cancer cells	90 % apoptosis, anti-angiogenesis	LncRNA delivery, combinatorial strategy	Requires genetic modulation	^60^
PLA-NPs	CDDP + CQ	OSCC Cal-27 cells	Induced apoptosis, ROS	Dual targeting, anti-autophagy	Chloroquine resistance risk	^61^
PDA@CUR@PCL/PLA	Curcumin	Oral squamous cell carcinoma (*In vitro* and *In vivo* model)	Photothermal elimination, no toxicity	pH/heat responsive	Photothermal equipment required	^62^
mPEG-PLA-OA Micelles	DOX	FaDu (HTB-43) cell line and 3D cultures model	Stronger apoptosis, deeper penetration	Improved tumor accumulation	Low loading efficiency	^63^
PLGA-NPs	Paclitaxel	FaDu cells	10x drug release, reduced IC50	Ultra-small, good permeability	Poor drug solubility	^64^
HA/CS-PLGA NPs	PTX + TMZ	CAL 27 cell line	Enhanced ROS, dual delivery	CD44 targeting, dual action	Limited *in vivo* validation	^65^
HA–PLGA-Ru NPs	Ru complex	TR146 cell line	>90 % cytotoxicity, PDT agent	Light-triggered response	Light exposure required	^66^
CUR-LCNPs	Curcumin	HPV-positive (UM-SCC-47, UPCI-SCC-154, and UM-SCC-104) and HPV-negative (UM-SCC-3, UM-SCC-27, and UT-SCC-26A) HNSCC cell lines	92.69 % encapsulation, tumor inhibition	Effective across HPV statuses	Photodynamic setup required	^67^
PEG-PLA Micelles	mTHPP	HSC-3 and HN-5 cancer cells	>85 % encapsulation, low toxicity	PDT suitable, solubility improved	Needs light source	^68^
Polymeric nanoparticles based on PEG–α-tocopheryl succinate (α-TOS)	Paclitaxel (PTX)	FaDu hypopharyngeal squamous cell carcinoma xenograft in mice	PTX-NPs reduced tumor volume, increased apoptosis, elevated oxidative/nitrosative stress; decreased Ki-67, EGFR, CD31, CD34, and Factor VIII expression	Dual-functional (carrier + therapeutic); Enhanced antitumor efficacy; Reduced systemic toxicity; Effective intratumoral delivery	Intratumoral injection limits clinical applicability; No systemic or long-term toxicity data	^69^
Liposomes + SLNs + PEG	Irinotecan + miR-200	SAS cells	Enhanced uptake, apoptosis induction	Gene + chemo co-delivery	Stability concerns	^70^
ATNH PCEC NPs	Honokiol	HNE-1 cells and Nude mouse xenograft model	Tumor inhibition, metabolic suppression	Folate targeting	Limited to folate receptor + tumors	^71^
PCL-NPs	Cisplatin + Curcumin	Oral epidermal carcinoma cells	Synergistic anticancer effect	Good combination index	Hydrophobicity may hinder delivery	^72^
NP-427	PHT-427	Hypopharyngeal squamous cell carcinoma xenograft mouse model.	Angiogenesis inhibition, necrosis	PI3K pathway suppression	May need combination therapy	^73^
PLGA nanoparticles	Polydatin	DMBA-induced oral carcinogenesis	Reduced tumor incidence and burden; restored antioxidant enzymes; enhanced cytochrome P450, GST; normalized histopathology; apoptosis induction	Strong chemopreventive activity; improves antioxidant and detox enzyme levels; induces apoptosis; restores tissue integrity	No clinical translation yet	^74^
PLGA nanoparticles	Nedaplatin	DMBA-induced OSCC in Syrian hamsters	Inhibited tumor formation; improved histological architecture; induced apoptosis; inhibited Notch signaling	Targets multiple oncogenic and anti-apoptotic markers; enhances tumor specificity and apoptosis	Preclinical stage only	^75^
CS-PLGA nanoparticles	Proanthocyanidin	drug-resistant OSCC cells	Suppressed proliferation, migration, and invasion; downregulated MMP-2 and MMP-9; indicated potential in overcoming chemoresistance	Combats drug resistance; impairs metastasis; biocompatible nanocarrier	No *in vivo* confirmation	^76^
PLGA nanoparticles	Toosendanin (TSN)	OSCC cells, nude mice model	Induced apoptosis and S-phase arrest; modulated JAK/STAT and PI3K–Akt pathways; demonstrated *in vivo* antitumor efficacy and biosafety	Improved solubility and bioavailability; safe and effective *in vivo*	Limited to preclinical stage	^77^
PLGA nanoparticles	Paclitaxel (PTX)	pharyngeal carcinoma cells	10-fold increase in drug release over 72 h; 50 % reduction in cell viability; enhanced intracellular uptake due to nanoscale size and surface charge	Improved drug delivery; enhanced cytotoxicity	No *in vivo* data	^78^
PEG-PCL micelles	Icaritin	OSCC cells	Sustained-release profile; enhanced colloidal stability; prolonged drug exposure; showed anti-proliferative and pro-apoptotic activity	Overcomes poor solubility and bioavailability of icaritin; suitable for targeted therapy	Lack of *in vivo* validation	^79^
PLGA nanoparticles	Dexamethasone	5-FU induced oral mucositis in hamster model	Reduced mucosal inflammation; lowered oxidative stress; modulated signalling pathways	Alleviates side effects of chemotherapy; strong anti-inflammatory and antioxidant properties	Disease-targeted efficacy unknown	^80^
Dendrimers (G4-FA)	siVEGFA	Xenograft HN12 tumor mouse model of HNSCC	Tumor retention, anti-angiogenic	High gene specificity	Immune response risk	^83^^,^^84^
Dendrimers (FA-MTX)	Methotrexate	SCID mice bearing various UM-SCC	Tumor targeting, reduced toxicity	Specific to folate receptors	Systemic bioavailability issues	^85^
Gadolinium Micelles	Pt (TPNP) + Gd	Head and neck tumor xenografts using Gadolinium-labelled phosphorescent polymeric nanomicelles	Enhanced imaging + localization	Dual diagnostic/therapeutic use	Contrast agent accumulation risk	^86^
CUR-NMs Micelles	Curcumin	Cisplatin-resistant KB cancer cells	Effective in MDR cancer	PEG-based stealth	Stability may vary	^87^
Micelles	mTHPP	HSC-3 and HN-5 cells	>90 % phototoxicity	Potent under light	Light-dependent	^88^
Micelles	mTHPP	HN5 and H2009 cancer cell lines	High ^1O_2_ generation	High therapeutic index	Fluorescence fluctuation	^89^
PAMAM Dendrimer	shRNA targeting hTERT	oral cancer cells, *in vivo* xenograft model	Silenced hTERT gene; inhibited proliferation; induced apoptosis; reduced tumor growth in xenograft mice	RNAi-based gene silencing; specific targeting of telomerase; effective tumor growth inhibition	Potential off-target effects; early-stage data	^90^
mPEG-PLA-OA Micelles	Doxorubicin + OA	oral cancer cells, 3D spheroids	Sustained release; enhanced cytotoxicity and apoptosis; superior tumor penetration; prolonged circulation; synergistic anticancer effects from OA	Active targeting via OA; improved stability and bioavailability; reduced systemic toxicity	Complex synthesis; needs clinical validation	^91^
CD44-targeted Micelles (mP6/Rg3)	Ginsenoside Rg3	oral CSCs), OSCC model	Inhibited ABCB1 and GPX4; altered ferroptosis gene expression (SLC7A11, FTH1); induced lipid peroxidation and ferroptosis; reduced stemness markers (CD44, ALDH1A1, SOX2); reduced CSC frequency and tumor growth	Targets CSCs; promotes ferroptosis; dual targeting of CD44 and ABCB1; reduces drug resistance	Specialized formulation; preclinical stage	^92^
PEG-ODA Micelles	Paclitaxel (PTX)	CAL-27 oral cancer cells mice bearing CAL-27 xenograft tumors	Stable formulation; internalized efficiently; 72 % tumor inhibition; no systemic toxicity; inhibited proliferation; favorable biosafety profile	Enhances PTX solubility; efficient tumor targeting; biocompatible and non-toxic nanocarrier	Long-term effects unknown	^93^

## 7 Clinical trial

Several clinical trials are exploring polymeric micelle-based nanomedicines in HNSCC and related cancers (Table 2). The Phase II trial NCT06366945 (not yet recruiting) evaluates neoadjuvant PM-PTX, carboplatin, and tislelizumab in resectable, lymph node-positive HNSCC to enhance major pathological response. Another ongoing Phase II trial, NCT06301165, compares the TPC regimen (PM-PTX + cisplatin + capecitabine) with gemcitabine–cisplatin in high-risk nasopharyngeal carcinoma focusing on 2-year progression-free survival. The completed Phase II study NCT02639858 investigated docetaxel-loaded polymeric micelles in recurrent/metastatic HNSCC after platinum failure, but results remain unpublished. NCT03585673 is an ongoing Phase II study using NANOXEL M, a PEG-PLA-based docetaxel micelle formulation, in esophageal squamous cell carcinoma and potentially HNSCC with about 38 participants. A large observational trial, NCT04066335, includes 1498 participants to assess the real-world safety of Nanoxel M across solid tumors, including HNSCC, monitoring hypersensitivity reactions and adverse events. Additionally, NCT06199895, a recruiting Phase II trial, examines PM-PTX in taxane-resistant solid tumors including HNSCC, utilizing the enhanced permeability and retention effect for better tumor targeting. Together, these studies highlight the growing focus on polymeric micelle nanocarriers for improving drug delivery, efficacy, and safety in HNC.^94^ (Data collected from US clinical trial website: https://clinicaltrials.gov).

**Table 2:** Ongoing and completed clinical trials involving polymeric micelle-based nanomedicines in HNSCC.

Trial ID	Phase/Status	Formulation/Regimen	Cancer Type	Study Objective/Endpoint	Participants
NCT06366945	Phase II/Not yet recruiting	PM-PTX + Carboplatin + Tislelizumab	Resectable, lymph node-positive HNSCC	Evaluate neoadjuvant therapy to improve major pathological response (MPR)	Not specified
NCT06301165	Phase II/Ongoing	TPC (PM-PTX + Cisplatin + Capecitabine) vs. GP (Gemcitabine + Cisplatin)	High-risk, locoregionally advanced NPC	Compare 2-year progression-free survival	Not specified
NCT02639858	Phase II/Completed (Results unpublished)	Docetaxel-loaded polymeric micelles (Docetaxel PM)	Recurrent/Metastatic HNSCC (post-platinum failure)	Assess efficacy and safety of Docetaxel PM	Not specified
NCT03585673	Phase II/Ongoing	NANOXEL M (PEG-PLA-based Docetaxel micelles)	Esophageal squamous cell carcinoma, potential HNSCC	Evaluate efficacy and safety	∼38
NCT04066335	Observational/Ongoing	Nanoxel M (Polymeric paclitaxel micelles)	Various solid tumors, including HNSCC	Monitor real-world safety, hypersensitivity reactions, and adverse events	1498
NCT06199895	Phase II/Recruiting	PM-PTX	Taxane-resistant solid tumors, including HNSCC	Assess efficacy and safety leveraging EPR effect for better drug targeting	Not specified

Among all the nanosystems described, **polymeric micelles hold greater translational impact,** as evidenced by their presence in multiple ongoing and completed clinical trials. Their ability to encapsulate poorly soluble chemotherapeutic drugs, improve tumor-specific delivery via the EPR effect, and combine with immunotherapeutics makes them highly promising for real-world clinical applications.

## 8 Conclusion and future perspective

Polymeric nanoparticles are a viable strategy for treating HNC because they allow for tailored drug administration, reduce systemic toxicity, and improve therapeutic effectiveness. Their ability to encapsulate chemotherapeutic agents and biomolecules ensures improved bioavailability and controlled release, addressing limitations of conventional treatments. Advances in surface modification further enable active targeting of tumor cells, offering precision in treatment. However, challenges such as scalability, long-term safety, and regulatory hurdles remain. Future research should focus on optimizing nanoparticle formulations for clinical translation, integrating imaging and therapy (theranostics), and personalizing treatment through molecular profiling. Looking ahead, the integration of polymeric nanoparticles with personalized medicine, immunotherapy, and real-time imaging holds exciting potential to enhance treatment outcomes further. Continued research into stimuli-responsive polymers, active targeting ligands, and multifunctional platforms will be key in advancing clinical translation. With ongoing innovation and clinical validation, polymeric nanoparticles are poised to play a transformative role in the precise and effective management of HNCs, offering renewed hope for improved patient survival and quality of life.

## Patient's/guardian's consent

Not Applicable.

## Ethics approval and consent

Not Applicable.

## Data availability statement

The author confirms that all data generated or analyzed during this review are included in this published article.

## Sources of funding

Authors have not received any financial support or funding for this review.

## Declaration of competing interest

The authors declare that they have no known competing financial interests or personal relationships that could have appeared to influence the work reported in this paper.
